# Assessing Lower-Limb Prosthetic Users with the Trinity Amputation and Prosthesis Experience Scale-Revised: A Cross-Sectional Study

**DOI:** 10.3390/jcm15031291

**Published:** 2026-02-06

**Authors:** Huthaifa Atallah, Amneh Alshawabka, Mahmoud Alfatafta, Tariq Alkhatib, Marwan Taher, Wafaa Saqer, Naqaa Obaidat, Hadeel R. Bakhsh, Anthony McGarry, Bálint Molics

**Affiliations:** 1 Department of Prosthetics and Orthotics, School of Rehabilitation Sciences, The University of Jordan, Amman 11942, Jordan; a.alshawabka@ju.edu.jo (A.A.); m.alfatafta@ju.edu.jo (M.A.); 2 Department of Physical Medicine & Rehabilitation, Al Basheer Hospitals Complex, Amman 11118, Jordan; tareq_alkhateeb@hotmail.com (T.A.); marwantah@yahoo.com (M.T.); 3 Remote Digital for Medical Equipment Trading LLC, Amman 11181, Jordannaqaaob@gmail.com (N.O.); 4 Department of Rehabilitation Sciences, College of Health and Rehabilitation Sciences, Princess Nourah bint Abdulrahman University, Riyadh 11564, Saudi Arabia; hrbakhsh@pnu.edu.sa; 5 Biomedical Engineering Department, University of Strathclyde, Glasgow G4 0NW, UK; anthony.mcgarry@strath.ac.uk; 6 Department of Sport Physiotherapy, Faculty of Health Sciences, University of Pécs, 7621 Pécs, Hungary; molics.balint@etk.pte.hu

**Keywords:** lower-limb amputation, prosthesis satisfaction, psychosocial adjustment, activity restriction, TAPES-R, rehabilitation

## Abstract

**Background:** Lower limb amputation affects physical function, mental health, and body image. Rehabilitation outcomes depend on both psychological adjustment and functional performance, including mobility and prosthesis satisfaction. This study aimed to evaluate psychological adjustment, activity restriction, and prosthesis satisfaction among lower-limb prosthetic users in Jordan using the Arabic TAPES-R. **Objective:** To assess, using a validated tool, the psychological adjustment, activity restriction, and prosthesis satisfaction of lower-limb prosthetic users in Jordan, aligning with the study title and cross-sectional design. **Methods:** This cross-sectional study included 74 unilateral lower-limb prosthetic users (66.2% male, mean age 42.4 ± 13.1 years). Sociodemographic and body composition characteristics were recorded. Participants completed the Arabic TAPES-R between September 2024 and April 2025. The TAPES-R measured psychosocial adjustment, activity restriction, and prosthesis satisfaction. Data were analyzed using descriptive statistics, Cronbach’s alpha for internal consistency, independent-samples *t*-tests, and Pearson correlations (*p* < 0.05). **Results:** Participants demonstrated generally positive psychosocial adjustment (Psychosocial Total = 3.08 ± 0.52) and moderate prosthesis satisfaction (Total Satisfaction = 2.23 ± 0.47), with variable activity restriction (8.76 ± 5.61). Internal consistency was strong across TAPES-R subscales (α = 0.816–0.955). Functional Satisfaction was higher in those with transfemoral than transtibial amputation (*p* = 0.041). Psychosocial adjustment correlated positively with prosthesis satisfaction (r = 0.48, *p* < 0.001) and negatively with activity restriction (r = −0.52, *p* < 0.001). Residual limb pain (45.9%) was associated with higher activity restriction (*p* = 0.022), and phantom limb pain (55.4%) with lower prosthetic satisfaction (*p* = 0.031). **Conclusions:** The Arabic TAPES-R effectively identifies psychological adjustment, activity restriction, and prosthesis satisfaction in lower-limb prosthetic users in Jordan. Participants generally reported positive psychosocial adjustment and moderate prosthesis satisfaction, but functional limitations remain, particularly in those with residual or phantom limb pain. These findings support the use of the TAPES-R as a clinical and research screening tool and provide guidance for targeted rehabilitation interventions.

## 1. Introduction

Globally, over 40 million people live with limb loss, with lower-limb amputation accounting for the majority of cases. The main clinical manifestations include impaired mobility, reduced independence, chronic pain, and psychological challenges such as depression, anxiety, and altered body image. The incidence of lower-limb amputation is increasing due to trauma, diabetes, vascular disease, and cancer, making it a significant public health concern worldwide.

The impact of lower limb amputation extends beyond physical impairment, affecting mental health, emotional well-being, and body image. Psychosocial adjustment following amputation is a dynamic process in which individuals learn to cope mentally, socially, and emotionally with limb loss [1,2,3]. Previous literature highlights quality of life (QoL), mobility, body image, and prosthesis satisfaction as key psychological factors influencing successful adjustment [4,5,6,7,8,9,10]. Mobility is particularly important, as increased mobility is strongly associated with greater independence, improved well-being, and higher prosthesis satisfaction [11,12]. Prosthetic appearance also influences satisfaction, as many individuals value the cosmetic aspects of their prosthesis as much as its functional performance [13]. Prostheses vary widely in design, including passive cosmetic limbs, body-powered prostheses, and advanced microprocessor-controlled or energy-storing devices. User acceptance depends on both biomechanical efficiencies such as gait stability, weight distribution, energy expenditure, and aesthetic considerations, including natural appearance, symmetry, and social confidence. Studies show that while advanced prostheses may improve functional outcomes, user satisfaction is also heavily influenced by cosmetic appearance and social perception [12,13,14]. Body image concerns are common after amputation, with many individuals reporting reduced self-esteem or discomfort with their appearance [15,16]. These findings underscore the importance of using assessment tools that capture emotional and psychosocial experiences alongside physical outcomes [17,18]. Following limb amputation, individuals often experience limitations in their activity levels, commonly related to pain, residuum condition, mobility, and prosthetic comfort [11,19]. Evidence suggests that advancements in prosthetic design can improve balance, gait, and patient satisfaction, thereby reducing functional limitations [14].

Despite these advancements, there remains limited research in Arabic-speaking countries, particularly studies that link psychosocial adjustment, functional outcomes, prosthesis satisfaction, and pain-related factors. Existing studies have primarily focused on validation of assessment tools or isolated outcomes, leaving gaps in understanding how these factors interact and influence rehabilitation success. By addressing these gaps, the present study contributes to the current state of the art by providing empirical data on the relationships between psychological adaptation, functional limitations, and prosthesis satisfaction in Jordanian lower-limb prosthesis users.

Several functional assessment scales are available to evaluate activity and mobility outcomes in individuals with amputation. Tools such as the Patient-Specific Functional Scale and the Lower Extremity Functional Scale are effective in tracking functional improvements during rehabilitation and assessing functional limitations [20,21]. Other measures, including the Prosthesis Evaluation Questionnaire–Mobility Subscale (PEQ-Mobility) and the Amputee Mobility Predictor, provide accurate assessments of mobility capabilities [22]. Importantly, mobility and activity levels are also influenced by psychological factors, such as pain self-efficacy, highlighting the complex interrelationship between psychological well-being and physical function restoration [23].

Satisfaction with the prosthesis is a critical determinant of rehabilitation success and has a significant impact on mobility, well-being, and quality of life [19]. Prosthesis satisfaction is influenced by several factors, including comfort, fit, alignment, cosmesis, durability, and functional performance during daily activities [13,24]. Studies have shown that residuum pain, prosthetic alignment, and durability play important roles in determining patient satisfaction [19,24]. Research on advanced prosthetic technologies demonstrates improvements in both mobility and satisfaction, reinforcing their value in prosthetic rehabilitation [14].

A validated instrument designed to assess these domains is the Trinity Amputation and Prosthesis Experience Scales (TAPES), which has demonstrated reliability in measuring psychosocial adjustment, activity restriction, and satisfaction with the prosthesis [17,18]. TAPES also provides comprehensive approaches to evaluating prosthesis satisfaction by incorporating both psychological and functional dimensions [17,18]. A study has validated the TAPES for Arabic-speaking prosthetic users [25]. However, while this study focused on validating and assessing the reliability of the TAPES, it did not perform any statistical analysis of the relevant variables. Consequently, evidence regarding the relationships between psychosocial adjustment, functional outcomes, and pain-related factors among Arabic-speaking prosthesis users remains limited.

Despite the growing body of international literature, there remains a lack of research focusing on psychological and functional outcomes among individuals with amputation in Arabic-speaking countries [25,26,27]. Given the increasing incidence of lower limb amputation in Jordan and the limited use of validated, culturally appropriate assessment tools, there is a clear need for further research in this context [28,29]. Few studies have examined emotional adjustment, activity restriction, and prosthesis satisfaction among Jordanian individuals with lower limb amputation. In particular, there is limited empirical evidence examining how pain-related factors may relate to these outcomes within this population. Addressing this gap may help inform more contextually appropriate rehabilitation strategies. The present study builds upon this prior validation work by moving beyond psychometric evaluation to examine associations between psychosocial adjustment, functional outcomes, prosthesis satisfaction, and pain-related factors. By focusing on outcome relationships, this study provides additional insight into the lived experiences of lower-limb prosthesis users within an Arabic-speaking context. Therefore, the aim of this study was to assess psychological adjustment, activity restriction, and prosthesis satisfaction in lower-limb prosthetic users in Jordan using the Arabic Trinity Amputation and Prosthesis Experience Scale-Revised (TAPES-R). The residual limb pain and phantom limb pain, and other medical problems were also assessed. The main hypotheses of the study were that higher psychosocial adjustment would be associated with lower activity restriction and greater prosthesis satisfaction, and that residual limb and phantom limb pain would negatively influence functional and satisfaction outcomes.

## 2. Materials and Methods

### 2.1. Study Design

This cross-sectional observational study was conducted at the Department of Physical Medicine and Rehabilitation, Al-Bashir Hospital, Amman, Jordan, to assess psychosocial adjustment, activity restriction, and satisfaction among lower-limb prosthesis users using the Arabic version of the Trinity Amputation and Prosthesis Experience Scales–Revised (TAPES-R).

### 2.2. Ethical Approval

Ethical approval was obtained from the Jordanian Ministry of Health Institutional Review Board (IRB number: 470/2024). Written informed consent was obtained from all participants prior to data collection, and participation was entirely voluntary.

### 2.3. Participants

**Figure 1:**
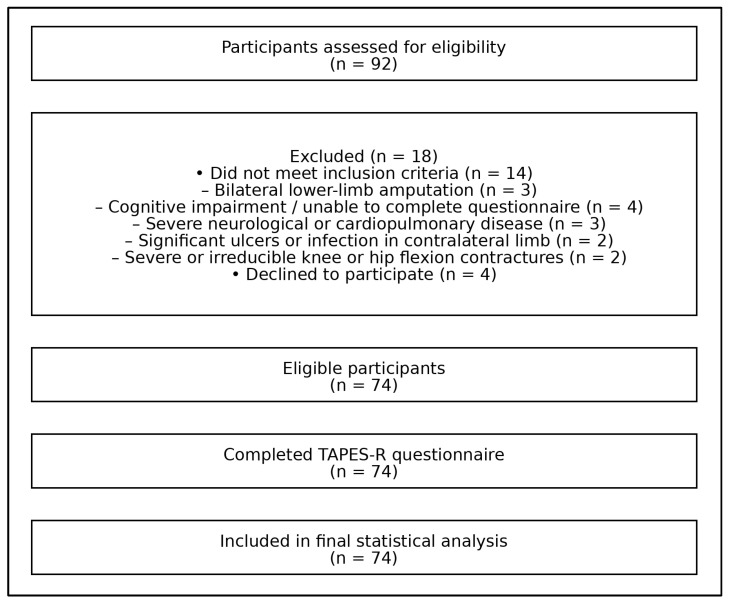
Flowchart illustrating participant recruitment, exclusion, and inclusion in the final statistical analysis.

Participants were recruited from the Department of Physical Medicine and Rehabilitation at Al-Bashir Hospital in Amman, Jordan. Inclusion criteria were: (1) adults (≥18 years), (2) People with unilateral lower-limb amputation who had been current prosthesis users for at least 3 months, (3) native Arabic speakers, (4) demonstrated good cognitive ability sufficient to independently understand and complete the questionnaire, (5) had at least one functional upper and lower limb, (6) were able to follow verbal commands and walk pain-free with their prosthesis, and (7) were not currently enrolled in a rehabilitation program at the time of data collection. Exclusion criteria included those with bilateral amputation, cognitive impairment, or any condition that interfered with independent questionnaire completion, advanced neurological or severe cardiopulmonary disease, significant ulcers or infections in the contralateral limb, or irreducible or severe knee or hip flexion contractures. A total of 74 participants met the inclusion criteria and completed the study. Data was collected using paper versions of the questionnaire between November 2024 and April 2025. All participants completed the questionnaires in person at the hospital, and no online or remote data collection methods were used. The process of participant recruitment, screening, exclusion, and final inclusion in the statistical analysis is summarized in Figure 1. A total of 92 individuals were assessed for eligibility, of whom 18 were excluded for not meeting the inclusion criteria or declining participation. The remaining 74 participants completed the TAPES-R questionnaire and were included in the final statistical analysis (Figure 1).

### 2.4. Procedure

The Arabic version of the Trinity Amputation and Prosthesis Experience Scales–Revised (TAPES-R), previously validated by Massarweh and Sobuh [25], was used in this study. The questionnaire consists of three main sections, psychosocial adjustment, activity restriction, and satisfaction, in addition to a minor section addressing residual limb pain, phantom limb pain, and other medical problems.

The psychosocial adjustment section included three subscales: general and social adjustments with five items for each, and adjustment to limitation with five items that were reverse scored. Items were rated on a four-point Likert scale. For the general adjustment and social adjustment subscales, response options ranged from one strongly disagree to four strongly agree. The range of each item score was categorized into strongly disagree (1.00–1.75), disagree (1.76–2.50), agree (2.51–3.25), and strongly agree (3.26–4.00). For the adjustment to limitation subscale, the response options ranged from one strongly agree to four strongly disagree. The range of each item score was categorized into strongly agree (1.00–1.75), agree (1.76–2.50), disagree (2.51–3.25), and strongly disagree (3.26–4.00). Each subscale score was calculated as the mean of its component items. Higher scores indicated better psychosocial adjustment (Supplementary Table S1).

The activity restriction section consisted of ten items rated on a three-point scale, zero not limited, one limited a little, and two limited a lot. Total scores ranged from zero to twenty, with higher scores indicating greater activity limitation. The range was categorized into not limited (0.00–6.66), limited a little (6.67–13.33), and limited a lot (13.34–20.00), with higher scores indicating lower activity limitation (Supplementary Table S1).

The satisfaction section included two subscales, aesthetic satisfaction with three items and functional satisfaction with five items. Items were scored on a three-point scale, one dissatisfied, two satisfied, and three very satisfied. The range of each item score was categorized into: not satisfied (1.00–1.66), satisfied (1.67–2.33), and very satisfied (2.34–3.00). Higher scores indicated greater satisfaction with the prosthesis (Supplementary Table S1).

The final section assessed the presence of phantom limb pain, residual limb pain, and any other medical problems not related to the amputation or prosthesis. Participants completed the questionnaire in person during follow-up visits at the rehabilitation clinic at the hospital. A trained researcher was available to clarify any questionnaire item as required. The average completion time was approximately ten to fifteen minutes.

**Figure 2:**
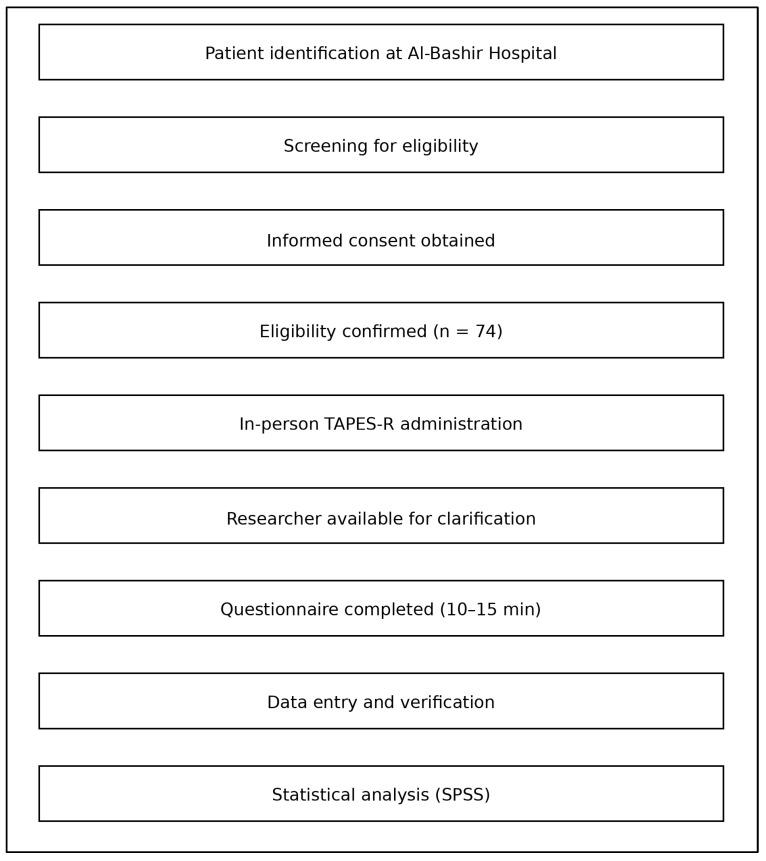
Graphic representation of the study logistics, from recruitment and screening to questionnaire administration and inclusion in the final analysis.

The overall study logistics, from participant recruitment through questionnaire administration and data collection, are illustrated in Figure 2. This figure provides a visual summary of the study workflow, including screening, eligibility assessment, in-person completion of the TAPES-R during follow-up visits, and inclusion in the final statistical analysis (Figure 2).

### 2.5. Statistical Analysis

All statistical analyses were performed using IBM SPSS Statistics, Version 22.0 (IBM Corp., Armonk, NY, USA). A priori sample size calculation was not performed because this was an observational, cross-sectional study with a fixed and limited population of lower-limb prosthesis users attending the rehabilitation clinic during the study period. Instead, all eligible and consenting participants who met the inclusion criteria were recruited consecutively. The final sample size of 74 participants was considered sufficient to provide stable estimates for descriptive statistics, internal consistency analysis, group comparisons, and correlation analyses, consistent with sample sizes used in similar prosthetics and rehabilitation studies.

Normality of continuous variables was assessed using the Shapiro–Wilk test and informed the selection of parametric procedures.

Descriptive statistics (means, standard deviations, frequencies, and percentages) were calculated for all demographic variables, TAPES-R subscales, and pain-related measures. Sociodemographic and clinical characteristics were collected for all participants using a structured data collection form administered in person. The following variables were recorded: gender, age (years), cause of amputation, level of amputation, time since amputation (years), and duration of prosthesis use (years).

Internal consistency of the TAPES-R subscales was evaluated using Cronbach’s alpha coefficients. Group differences between those with transtibial and transfemoral amputation were examined using independent-samples *t*-tests, as all TAPES-R subscales met normality assumptions. Formal effect size indices were not calculated; however, the magnitude of group differences and associations was interpreted using the corresponding test statistics and correlation coefficients. Pearson correlation coefficients were used to assess relationships between continuous variables, including time since amputation, TAPES-R subscale scores, and pain characteristics (such as pain intensity and interference). Assumptions for Pearson correlation analyses were examined prior to analysis. Linearity was assessed through visual inspection of scatterplots, and no significant outliers were identified. A two-tailed significance level of *p* < 0.05 was considered statistically significant.

## 3. Results

### 3.1. Demographic Characteristics

The study included 74 prosthesis users with a mean age of 42.4 ± 14.7 years (range 18–75). Most participants were male (66.2%). Trauma was the most common cause of amputation (32.4%), followed by diabetes (14.9%) and cancer-related etiologies (14.9%). The majority of participants had transtibial amputations (56.8%), while 33.8% had transfemoral amputations (Table 1).

The mean time since amputation was 15.1 ± 11.7 years (range 2–30), and the mean duration of prosthetic use was 8.8 ± 7.7 years (range 1–29). Collectively, these data represent a heterogeneous and clinically representative cohort of adult lower-limb prosthesis users.

### 3.2. TAPES-R Outcomes

Participants demonstrated generally positive psychosocial adaptation to limb loss. High mean scores were observed for General Adjustment (3.33 ± 0.55 out of 4) and Social Adjustment (3.32 ± 0.77 out of 4), both corresponding to a strongly agree level. Adjustment to Limitation showed a comparatively lower mean score (2.50 ± 0.82 out of 4), indicating disagreement. The overall Psychosocial Adjustment total score was 3.08 ± 0.52 out of 4, reflecting an overall agree level. Activity Restriction scores ranged from 0 to 20, with a mean of 8.76 ± 5.61, corresponding to being “limited a little”. Overall satisfaction with the prosthesis was moderate to high. Aesthetic Satisfaction demonstrated a higher mean score (2.34 ± 0.55 out of 3), indicating participants were very satisfied, while Functional Satisfaction showed a slightly lower mean score (2.16 ± 0.54 out of 3), reflecting satisfaction. The total Satisfaction score was 2.23 ± 0.47 out of 3, indicating that participants were generally satisfied with their prosthetic devices (Table 2).

No significant differences were observed between individuals with transtibial and transfemoral amputations across most TAPES-R subscales. However, Functional Satisfaction was significantly higher in the transtibial group (2.34 ± 0.48) compared with the transfemoral group (2.07 ± 0.57; *p* = 0.041) (Supplementary Table S2). Residual limb pain was linked to activity restriction, phantom limb pain to lower prosthesis satisfaction, and psychosocial adjustment showed only weak associations with pain.

Longer time since amputation was significantly associated with higher General Adjustment (r = 0.497, *p* < 0.001), Adjustment to Limitation (r = 0.327, *p* = 0.006), Psychosocial Total (r = 0.389, *p* = 0.001), and Functional Satisfaction (r = 0.406, *p* < 0.001). Time since amputation was negatively correlated with Activity Restriction (r = −0.247, *p* = 0.039). Correlations are summarized in (Supplementary Table S3). Longer time since amputation was associated with higher General Adjustment, Adjustment to Limitation, Psychosocial Total, and Functional Satisfaction, and with lower Activity Restriction. Other subscales, including Social Adjustment and Aesthetic Satisfaction, showed no significant correlations.

All TAPES-R subscales demonstrated strong reliability with Cronbach’s alpha coefficients ranging from 0.816 to 0.955 (Supplementary Table S4). All TAPES-R subscales demonstrated strong internal consistency, with Cronbach’s alpha coefficients ranging from 0.816 for Adjustment to Limitation to 0.955 for Aesthetic Satisfaction, indicating high reliability across psychosocial, activity, and satisfaction domains. Significant correlations were observed among TAPES-R domains. Psychosocial Total was moderately negatively correlated with Activity Restriction (r = −0.39) and moderately positively correlated with Total Satisfaction (r = 0.40). Aesthetic and Functional Satisfaction were strongly correlated (r = 0.44), as were Functional and Total Satisfaction (r = 0.92) (Supplementary Table S5). Psychosocial Total was moderately negatively correlated with Activity Restriction and moderately positively correlated with Total Satisfaction. Strong correlations were observed between Aesthetic and Functional Satisfaction, and between Functional and Total Satisfaction, highlighting close relationships among satisfaction subscales.

### 3.3. Pain Experiences

Residual limb pain and phantom limb pain were both common in this cohort. Overall, 34 participants (45.9%) reported residual limb pain, while 41 (55.4%) reported phantom limb pain. Additionally, 22 participants (29.7%) reported other medical problems not directly related to amputation-site or phantom pain (Table 3).

Among those with residual limb pain (*n* = 34), the most frequently reported intensity category was “unpleasant pain” (55.9%), followed by “mild pain” (20.6%). More severe categories were less common, with “terrible pain” reported by 14.7%, “excruciating pain” by 5.9%, and “frustrating pain” by 2.9% of those affected (Table 4).

Regarding the impact of residual limb pain on daily life, 44.1% of participants with residual limb pain described a “decent effect” on activities, and a further 23.6% reported “significant impact” or “slight” effect. In contrast, only 14.7% indicated that residual limb pain had no effect or “absolutely no effect” on their work, social, or family activities. Participants with residual limb pain reported significantly greater activity restriction compared to those without residual limb pain (mean Activity Restriction 10.44 vs. 7.33, *p* = 0.017), while psychosocial adjustment and satisfaction scores were not significantly different (Table 5).

Phantom limb pain was slightly more prevalent than residual limb pain, reported by 41 participants (55.4%)**.** Among those experiencing phantom pain, intensity was most often rated as “unpleasant pain” (41.5%) or “mild pain” (34.1%), with more severe categories such as “excruciating” and “terrible” pain comprising a smaller proportion (approximately 17.1% combined). Only a minority described phantom pain as “frustrating” (Table 4).

Phantom limb pain also interfered with everyday activities to varying degrees. Among those reporting phantom pain, around half described the impact as “a little” or “slight” (both 24.4%), whereas 22.0% indicated a “decent effect” and 7.3% reported a “significant impact” on work, social, or family activities. Despite this, 22.0% indicated that phantom pain had “no” or “absolutely no” effect on their daily functioning (Table 5).

Phantom pain was not significantly associated with psychosocial adjustment or activity restriction. However, it was associated with lower prosthesis satisfaction, particularly in the functional domain: participants with phantom pain had significantly lower Functional Satisfaction scores than those without phantom pain (mean 2.00 vs. 2.36, *p* = 0.004), and lower Total Satisfaction (mean 2.12 vs. 2.36, *p* = 0.027).

No significant differences were observed between individuals with transtibial and transfemoral amputations in the prevalence or intensity of either residual limb pain or phantom limb pain. Both groups demonstrated comparable distributions across pain severity categories and reported similar levels of pain-related interference with daily activities. Longer time since amputation showed a general trend toward lower phantom limb pain intensity, reduced pain-related interference, and higher prosthesis satisfaction.

## 4. Discussion

This study used the Arabic version of the TAPES-R to comprehensively assess psychological adjustment, activity restriction, and prosthesis satisfaction among lower-limb prosthetic users in Jordan. Residual limb pain, phantom limb pain, and other medical problems were also evaluated. In addition, correlation analyses were conducted to examine the relationships among the study variables. The following sections interpret the findings not only from a clinical rehabilitation perspective, but also in relation to basic neuroanatomical and neurophysiological mechanisms underlying pain perception, sensorimotor integration, and psychological adaptation after limb loss.

### 4.1. Demographic Characteristics

The present study provides important insight into the demographic and clinical characteristics of lower-limb prosthetic users in Jordan (Table 1). The predominance of younger male participants likely reflects the higher exposure of working-age men in Jordan to traumatic risk factors, as well as their greater likelihood of seeking and continuing prosthetic rehabilitation services. This distribution is consistent with previous reports from Jordan, the Gulf region, and the broader Middle Eastern context, where males constitute the majority of individuals with limb loss, largely due to higher exposure to occupational hazards, road traffic accidents, and other traumatic events [9,30]. However, this demographic profile may not fully represent the overall population of prosthetic users in Jordan, particularly older individuals and those with dysvascular etiologies, who may be underrepresented in research studies.

The predominance of trauma-related amputations highlights the continued public health burden of road traffic accidents and occupational injuries in Jordan, while the presence of metabolic-related amputations reflects the growing impact of chronic disease. The high proportion of trauma-related amputations aligns with regional literature identifying trauma as a leading cause of limb loss among males in Middle Eastern populations [31]. At the same time, the notable presence of diabetic and vascular amputations is in agreement with national data from Jordan and international studies linking the rising prevalence of metabolic diseases to increasing rates of lower-extremity amputation [28,32]. The discrepancy between the present findings and those of Alfatafta et al. (2025), who reported diabetes mellitus as the leading etiology [33], may be explained by differences in sample characteristics, particularly the younger mean age in the current study. Individuals with traumatic amputations are generally younger, more physically active, and more likely to participate in studies involving functional or physical assessments compared with those with dysvascular amputations.

With respect to amputation level, transtibial amputation was substantially more prevalent than transfemoral amputation (56.8% vs. 33.8%). This finding is consistent with established clinical practice, where limb-salvage strategies prioritize preservation of the knee joint whenever possible due to its significant impact on gait efficiency, functional mobility, and overall prosthetic outcomes. The higher prevalence of transtibial amputations may therefore reflect both surgical decision-making aimed at optimizing function and the relatively younger, trauma-dominated profile of the study population.

Most participants were long-term post-amputation and long-term prosthesis users, suggesting substantial experience living with limb loss and using prosthetic devices. This extended time since amputation and prolonged duration of prosthesis use are important contextual factors when interpreting outcomes related to psychological adjustment, activity restriction, and prosthesis satisfaction. Previous research indicates that adaptation to limb loss and prosthetic use evolves over time, with long-term users often demonstrating better coping strategies, functional independence, and more stable perceptions of their prosthesis compared with recent prosthetic users. Consequently, the findings of the present study may primarily reflect the experiences of established prosthetic users rather than those in the early stages of rehabilitation.

Overall, the demographic and clinical profile observed in this study aligns with regional and international literature, while also highlighting population-specific characteristics of lower-limb prosthetic users in Jordan. These factors should be carefully considered when generalizing the results and when designing future studies or rehabilitation programs targeting more diverse age groups, etiologies, and stages post-amputation.

### 4.2. TAPES-R Outcomes

These findings suggest that long-term prosthesis users in this setting may develop effective emotional and social coping strategies, even in the presence of physical and environmental challenges (Table 2). Participants showed higher levels of general and social adjustment compared with adjustment to limitations, indicating that most individuals had emotionally accepted their limb loss and successfully reintegrated socially despite persisting functional and participation-related challenges. This finding suggests that psychological and social adaptation may occur independently of physical and environmental constraints, particularly among long-term prosthesis users. Similar patterns have been reported in previous studies using the TAPES and TAPES-R, which highlight the role of coping strategies, social support, and time since amputation in facilitating psychosocial adjustment beyond purely physical functioning [1,17,34,35].

In the Jordanian context, limitations related to activity and participation appear to be influenced by both time since amputation and underlying health conditions. Previous Jordan-based studies have shown that psychological outcomes following lower-limb amputation are shaped not only by physical impairments but also by the duration since amputation and the surrounding social context [29]. In the present study, activity restriction scores indicated a moderate level of participation limitation overall, with considerable inter-individual variability, most commonly reflecting being “limited a little.” This variability is consistent with earlier reports highlighting substantial differences in functional levels among lower-limb prosthesis users [19]. Such heterogeneity likely reflects the combined influence of multiple interacting factors, including clinical characteristics, level of amputation, prosthetic fit and componentry, access to and quality of rehabilitation services, presence of comorbidities, and individual coping strategies [11] (Table 2).

The higher emphasis on aesthetic satisfaction may reflect the importance of body image and social acceptance, whereas functional dissatisfaction likely reflects ongoing biomechanical, comfort, and performance challenges in daily life. This finding is consistent with previous studies emphasizing the importance of cosmetic appearance and social presentation for body image, self-esteem, and social confidence among individuals with lower-limb amputation [13,15,36]. In contrast, satisfaction with prosthetic function demonstrated greater variability, which may reflect differences in socket comfort, residuum health, individual adaptation, and the ability of the prosthesis to meet the demands of daily activities [19]. While dissatisfaction with prosthetic aesthetics has been reported elsewhere [13], the high proportion of long-term prosthesis users in the present study may partly explain the overall positive satisfaction levels, as prolonged use is often associated with improved prosthetic fit, enhanced functional adaptation, and more realistic user expectations over time [1,24] (Table 2).

When outcomes were compared based on the level of amputation, no significant differences were observed between transtibial and transfemoral users in terms of psychosocial adjustment, activity limitation, or overall prosthesis satisfaction, although transfemoral users reported higher functional satisfaction (Supplementary Table S2). This finding aligns with previous literature suggesting that rehabilitation outcomes and satisfaction are influenced by a complex interplay of factors including prosthetic fit, residuum health, rehabilitation intensity, and access to services rather than amputation level alone [19]. The results are also consistent with the design of the TAPES-R, which emphasizes the user’s lived experience and adaptation over purely biomechanical considerations [17,18]. The relatively higher functional satisfaction among transfemoral users, despite greater physiological demands, may reflect a recalibration of expectations or a response shift, whereby functional improvements are evaluated relative to a lower pre-prosthetic baseline [1]. Additionally, it is possible that individuals with transfemoral amputation participate in more intensive rehabilitation programs, which have been shown to positively influence perceived function and satisfaction [19]. These findings support the idea that adaptation following amputation may be a gradual process involving the development of coping strategies, acquisition of new skills, and re-establishment of personal identity over time, but causal conclusions cannot be drawn.

Longer time since amputation was associated with better psychosocial adjustment, higher overall prosthesis satisfaction particularly functional satisfaction and lower levels of activity restriction, although the relationship with social adjustment was weaker (Supplementary Table S3). These findings support the concept that adaptation following amputation is a gradual process involving the development of coping strategies, acquisition of new skills, and re-establishment of personal identity over time [1]. Evidence from Jordan similarly indicates that psychological distress is typically higher in the early post-amputation period and decreases as individuals adjust over time [29]. Longitudinal studies of trauma-related individuals with lower limb amputation further suggest that prolonged prosthesis use is associated with increased satisfaction and functional stability as individuals gain experience and optimize prosthetic use [24]. The relatively weaker association between time since amputation and social adjustment may reflect the greater impact of external contextual factors such as employment status, family and community support, societal attitudes, and environmental accessibility on social reintegration, rather than time alone.

Correlation analyses between psychosocial adjustment, activity restriction, and prosthesis satisfaction (Supplementary Tables S4 and S5) revealed that while these domains are interrelated, they represent distinct aspects of the prosthesis experience. Higher psychosocial adjustment was associated with lower activity restriction and greater prosthesis satisfaction, suggesting that individuals who adapt more effectively emotionally and socially tend to report higher participation levels and more positive prosthesis experiences. This finding aligns with biopsychosocial models of limb loss, which emphasize the interplay between psychological adaptation and engagement in daily life [1]. The observed relationship also supports the TAPES-R framework, which conceptualizes prosthesis experience as a multidimensional but interconnected construct encompassing emotional, social, and functional domains [17,18]. However, activity restriction demonstrated a weaker correlation with prosthesis satisfaction, indicating that satisfaction with the device does not necessarily translate into unrestricted participation. This is consistent with evidence showing that prosthesis satisfaction is often driven by device-related factors such as aesthetics, comfort, and fit, whereas participation outcomes are more strongly influenced by health status, comorbidities, and environmental barriers [19].

### 4.3. Pain Experience

Pain remains a prominent concern among unilateral lower-limb prosthesis users in the present study. The persistence of pain highlights the chronic nature of post-amputation discomfort and underscores the need for long-term pain management strategies integrated within rehabilitation services (Table 3). In addition, approximately one-third of the sample reported other medical conditions, underscoring the substantial burden of comorbidities within this population. These findings are consistent with previous literature demonstrating that post-amputation pain is widespread and represents a significant clinical challenge that negatively affects functional outcomes and overall quality of life among individuals with lower-limb amputation [37,38].

Analysis of pain intensity revealed that most participants described their residual limb pain (55.9%) and phantom limb pain (41.5%) as unpleasant, while only a small minority reported frustrating levels of pain for residual limb pain (2.9%) and phantom limb pain (4.9%) (Table 4). Pain was also reported to influence daily activities (Table 5). Participants with residual limb pain most frequently reported a moderate impact on daily activities (44.1%), whereas those with phantom limb pain demonstrated a more evenly distributed impact across all four activity limitation levels. This variability aligns with existing evidence indicating that pain experiences following amputation are heterogeneous, with only a subset of individuals experiencing severe and highly disabling pain [37]. One possible explanation is that the current sample consisted exclusively of ambulatory prosthesis users, as individuals experiencing severe pain are more likely to reduce prosthesis use or discontinue rehabilitation altogether [19,24]. Furthermore, the use of categorical pain descriptors rather than numerical rating scales may have influenced the distribution of pain severity, as prior research has shown that different pain assessment methods yield varying interpretations of pain burden in populations of those with amputation [37].

This relationship suggests that localized pain at the residuum directly interferes with functional performance by limiting tolerance to weight-bearing and prosthetic loading. These associations are observational and should not be interpreted as causal due to the cross-sectional design of the study. This finding is consistent with earlier studies demonstrating that residual limb pain reduces load tolerance, limits weight-bearing capacity, and consequently impairs functional performance [19]. This pattern indicates that phantom limb pain may primarily affect perceived comfort and effort rather than actual participation, thereby influencing satisfaction more than activity engagement. Again, these relationships reflect correlations rather than causal effects. This suggests that while phantom limb pain may not prevent participation in daily activities, it negatively influences prosthetic comfort, perceived effort, and usability, thereby diminishing satisfaction despite maintained functional engagement [1,37].

Interestingly, neither residual limb pain nor phantom limb pain showed a strong association with overall psychosocial adjustment. These findings indicate correlation only and cannot establish causation due to the cross-sectional study design. This finding aligns with previous research from Jordan, indicating that psychological adjustment following amputation is more strongly influenced by social, environmental, and contextual factors than by pain alone [29]. Additionally, long-term prosthesis users may develop adaptive coping strategies that mitigate the psychological impact of persistent pain, thereby buffering its influence on psychosocial outcomes. The observed patterns should be interpreted as associations rather than proof of causal effects.

No significant differences in pain prevalence or intensity were observed between transtibial and transfemoral amputation levels. Although time since amputation demonstrated an inverse relationship with both phantom limb pain and phantom limb sensation, these associations did not reach statistical significance. As with all other findings, these relationships are correlational and cannot imply causation. This may be attributed to the relatively small subgroup sizes and the heterogeneity of the sample with respect to amputation etiology, prosthetic fit, pain management approaches, and rehabilitation quality. These factors likely exert a stronger influence on pain outcomes than amputation level alone, as supported by previous studies [19,38].

Overall, pain continues to represent a substantial challenge for unilateral lower-limb prosthesis users in Jordan. While psychosocial adjustment and prosthesis satisfaction were generally favorable, residual limb pain remained a key contributor to activity restriction, and phantom limb pain adversely affected functional satisfaction. These relationships reflect associations observed in this cross-sectional study and should not be interpreted as causal. These findings emphasize the necessity of integrating targeted pain management strategies within rehabilitation programs. Reliance solely on satisfaction or adjustment measures may overlook persistent pain-related limitations, highlighting the importance of comprehensive, multidimensional outcome assessment when evaluating rehabilitation success [11,19].

Future studies should consider longitudinal designs to track changes in psychological adjustment, activity restriction, and prosthesis satisfaction over time, particularly including individuals in the early post-amputation period. Intervention studies targeting pain management, functional training, and psychosocial support could provide insights into causal relationships and effective strategies to enhance prosthetic outcomes. Additionally, expanding the sample to include more diverse age groups, amputation etiologies, and rehabilitation settings would strengthen the generalizability of findings and help tailor rehabilitation programs to specific subgroups of lower-limb prosthesis users.

## 5. Conclusions

This study suggests that lower-limb prosthesis users in Jordan generally appear to exhibit positive psychosocial adjustment and overall prosthesis satisfaction when assessed using the Arabic version of the TAPES-R, which serves as a valuable clinical and research screening tool rather than a definitive outcome measure, particularly among long-term, ambulatory users. These findings may indicate successful emotional and social adaptation over time, despite ongoing functional challenges.

However, pain remains a significant issue. Residual limb pain was strongly associated with activity restriction, while phantom limb pain negatively associated with functional and overall prosthesis satisfaction without necessarily limiting participation. Notably, pain was not strongly linked to psychosocial adjustment, indicating that psychological adaptation could be influenced more by social and contextual factors than by pain alone.

Outcomes did not differ significantly by amputation level, supporting the view that rehabilitation success likely depends on multiple interacting factors beyond amputation level. Overall, these findings highlight the importance of a multidimensional rehabilitation approach that integrates targeted pain management with functional and psychosocial interventions to better address participation limitations and potentially optimize long-term outcomes for lower-limb prosthesis users in Jordan. Future longitudinal and interventional studies are needed to clarify causal relationships and improve rehabilitation strategies.

### Study Limitations

This study was conducted at a single institution with a moderate sample size, which may limit the generalizability of the findings to all lower-limb prosthesis users in Jordan and other Arabic-speaking populations. The inclusion of only unilateral, ambulatory users and the exclusion of individuals in active rehabilitation may have led to an underestimation of activity limitations, psychosocial burden, and pain severity. Additionally, reliance on self-reported data may have introduced recall and response bias. Furthermore, the study did not include longitudinal follow-up or objective functional measures (e.g., gait or mobility performance tests), which limits the ability to assess changes over time or validate self-reported functional outcomes.

## Figures and Tables

**Table 1:** Participant demographic characteristics (n. = 74).

Variable	Category/Statistic	No. (%) or Mean ± SD (Range)
Gender	Male	49 (66.2%)
	Female	25 (33.8%)
Age (years)	Mean ± SD	42.4 ± 14.7
	Range	18–75
Cause of amputation	Trauma/Accident	24 (32.4%)
	Diabetes	11 (14.9%)
	Cancer	11 (14.9%)
	Vascular disease	5 (6.8%)
	Infection/Inflammation	6 (8.1%)
	Congenital deformity	2 (2.7%)
	Other causes *	15 (20.3%)
Level of amputation	Below-knee (Transtibial)	42 (56.8%)
	Above-knee (Transfemoral)	25 (33.8%)
	Knee disarticulation	4 (5.4%)
	Hip disarticulation	1 (1.4%)
	Foot-level amputation	1 (1.4%)
	Hip joint-level amputation	1 (1.4%)
Time since amputation (years)	Mean ± SD	15.1 ± 11.7
	Range	2–30
Duration of prosthesis use (years)	Mean ± SD	8.8 ± 7.7
	Range	1–29

* Other causes include: war injuries, medical errors, tissue necrosis, nerve damage, spinal deformity complications, vascular ischemia, and other rare medical or surgical conditions.

**Table 2:** Descriptive Statistics for TAPES-R Subscales (*n* = 74).

TAPES-R Subscale		Mean ± SD	Range	Interpretation
Psychosocial adjustment	General Adjustment (out of 4)	3.33 ± 0.55	1.8–4.0	Strongly agree
	Social Adjustment (out of 4)	3.32 ± 0.77	1.0–4.0	Strongly agree
	Adjustment to Limitation (out of 4)	2.50 ± 0.82	1.0–4.0	Disagree
	Total score (out of 4)	3.08 ± 0.52	1.5–4.0	Agree
Activity Restriction (out of 20)		8.76 ± 5.61	0.0–20	Limited a little
Satisfaction with prosthesis	Aesthetic Satisfaction (out of 3)	2.34 ± 0.55	1.0–3.0	Very satisfied
	Functional Satisfaction (out of 3)	2.16 ± 0.54	1.0–3.0	Satisfied
	Total score (out of 3)	2.23 ± 0.47	1.0–3.0	Satisfied

**Table 3:** Prevalence of pain types (*n* = 74).

Pain Type	Category	*n*	%
Residual limb pain	Yes	34	45.9
Residual limb pain	No	40	54.1
Phantom limb pain	Yes	41	55.4
Phantom limb pain	No	33	44.6
Other medical problems (besides amputation-site/phantom pain)	Yes	22	29.7
Other medical problems (besides amputation-site/phantom pain)	No	52	70.3

**Table 4:** Intensity of pain among those with pain.

Intensity Category	*n*	%
Residual limb pain intensity (*n* = 34)		
Mild pain	7	20.6
Unpleasant pain	19	55.9
Terrible pain	5	14.7
Excruciating pain	2	5.9
Frustrating pain	1	2.9
Phantom limb pain intensity (*n* = 41)		
Mild pain	14	34.1
Unpleasant pain	17	41.5
Terrible pain	3	7.3
Excruciating pain	4	9.8
Frustrating pain	2	4.9

**Table 5:** Impact of pain on daily activities.

Intensity Category	*n*	%
Residual limb pain impact (*n* = 34)		
No effect/absolutely no effect	5	14.7
A little	6	17.6
Slight	4	11.8
Decent effect	15	44.1
Significant impact	4	11.8
Phantom limb pain impact (*n* = 41)		
No effect/absolutely no effect	9	22.0
A little	10	24.4
Slight	10	24.4
Decent effect	9	22.0
Significant impact	3	7.3

## Data Availability

The datasets used and/or analyzed during the current study are available from the corresponding author on reasonable request.

## Supplementary Materials

The following supporting information can be downloaded at: https://www.mdpi.com/article/10.3390/jcm15031291/s1, Supplementary Table S1: Scoring Ranges and Interpretation of TAPES-R Scores; Supplementary Table S2: TAPES-R Scores by Amputation Level; Supplementary Table S3: Relationship Between Time Since Amputation and TAPES Outcomes; Supplementary Table S4: Cronbach’s Alpha for TAPES-R Subscales; Supplementary Table S5: Correlation Matrix for TAPES-R Domains.

## Abbreviations

The following abbreviations are used in this manuscript:

TAPES-R	Trinity Amputation and Prosthesis Experience Scale-Revised
