# Supplementary material for: Assessing Lower-Limb Prosthetic Users with the Trinity Amputation and Prosthesis Experience Scale-Revised: A Cross-Sectional Study
Source: J Clin Med. 2026 Feb 6;15(3):1291. doi: 10.3390/jcm15031291 (PMC12898395; doi:10.3390/jcm15031291)
Supplement: Supplementary file 1 [file jcm-15-01291-s001.zip › Supplementary Table 5.pdf]

Supplementary Table 5: Correlation Matrix for TAPES-R Domains.

Subscale	PT	AR	AES	FNS	TS
Psychosocial Total (PT)	1.00	−0.39	0.49	0.25	0.40
Activity Restriction (AR)	−0.39	1.00	−0.06	−0.15	−0.14
Aesthetic Satisfaction (AES)	0.49	−0.06	1.00	0.44	0.76
Functional Satisfaction (FNS)	0.25	−0.15	0.44	1.00	0.92
Total Satisfaction (TS)	0.40	−0.14	0.76	0.92	1.00
