# Supplementary material for: Assessing Lower-Limb Prosthetic Users with the Trinity Amputation and Prosthesis Experience Scale-Revised: A Cross-Sectional Study
Source: J Clin Med. 2026 Feb 6;15(3):1291. doi: 10.3390/jcm15031291 (PMC12898395; doi:10.3390/jcm15031291)
Supplement: Supplementary file 1 [file jcm-15-01291-s001.zip › Supplementary Table 1.pdf]

Supplementary Table 1: Scoring Ranges and Interpretation of TAPES-R Scores

TAPES-R Subscale		Range	Interpretation
**Psychosocial adjustment**	**General Adjustment and Social Adjustment**	1.00 – 1.75	Strongly disagree
		1.76 – 2.50	Disagree
		2.51 – 3.25	Agree
		3.26 – 4.00	Strongly agree
	**Adjustment to Limitation**	1.00 – 1.75	Strongly agree
		1.76 – 2.50	Agree
		2.51 – 3.25	Disagree
		3.26 – 4.00	Strongly disagree
**Activity Restriction**		0.00 – 6.66	Limited
		6.67 – 13.33	Limited a little
		13.34 – 20.00	Limited a lot
**Satisfaction with prosthesis**	**Aesthetic Satisfaction and Functional Satisfaction**	1.00 – 1.66	Not satisfied
		1.67 – 2.33	Satisfied
		2.34 – 3.00	Very satisfied
