# Supplementary material for: Assessing Lower-Limb Prosthetic Users with the Trinity Amputation and Prosthesis Experience Scale-Revised: A Cross-Sectional Study
Source: J Clin Med. 2026 Feb 6;15(3):1291. doi: 10.3390/jcm15031291 (PMC12898395; doi:10.3390/jcm15031291)
Supplement: Supplementary file 1 [file jcm-15-01291-s001.zip › Supplementary Table 2.pdf]

***Supplementary Table 2: TAPES-R Scores by Amputation Level (NO = 74)***

| TAPES-R Subscale | | Transtibial (Mean ± SD) | Transfemoral (Mean ± SD) | p-value |
|---|---|---|---|---|
| **Psychosocial adjustment** | **General Adjustment (out of 4)** | 3.26 ± 0.58 | 3.39 ± 0.46 | 0.305 |
| | **Social Adjustment (out of 4)** | 3.31 ± 0.77 | 3.34 ± 0.76 | 0.854 |
| | **Adjustment to Limitation (out of 4)** | 2.41 ± 0.84 | 2.51 ± 0.74 | 0.588 |
| | **Total score (out of 4)** | 3.03 ± 0.52 | 3.13 ± 0.44 | 0.404 |
| **Activity Restriction (out of 20)** | | 9.76 ± 5.67 | 8.08 ± 5.30 | 0.226 |
| **Satisfaction with prosthesis** | **Aesthetic Satisfaction (out of 3)** | 2.34 ± 0.56 | 2.32 ± 0.56 | 0.881 |
| | **Functional Satisfaction (out of 3)** | 2.07 ± 0.57 | 2.34 ± 0.48 | **0.041** |
| | **Total score (out of 3)** | 2.17 ± 0.50 | 2.34 ± 0.43 | 0.161 |
