# Supplementary material for: Assessing Lower-Limb Prosthetic Users with the Trinity Amputation and Prosthesis Experience Scale-Revised: A Cross-Sectional Study
Source: J Clin Med. 2026 Feb 6;15(3):1291. doi: 10.3390/jcm15031291 (PMC12898395; doi:10.3390/jcm15031291)
Supplement: Supplementary file 1 [file jcm-15-01291-s001.zip › Supplementary Table 3.pdf]

Supplementary Table 3: Relationship Between Time Since Amputation and TAPES Outcomes

TAPES-R Subscale		r	p-value
Psychosocial adjustment	General Adjustment (out of 4)	0.497	<0.001
	Social Adjustment (out of 4)	0.070	0.564
	Adjustment to Limitation (out of 4)	0.327	0.006
	Total score (out of 4)	0.389	0.001
Activity Restriction (out of 20)		−0.247	0.039
Satisfaction with prosthesis	Aesthetic Satisfaction (out of 3)	0.159	0.189
	Functional Satisfaction (out of 3)	0.406	<0.001
	Total score (out of 3)	0.366	0.002
