# Supplementary material for: Assessing Lower-Limb Prosthetic Users with the Trinity Amputation and Prosthesis Experience Scale-Revised: A Cross-Sectional Study
Source: J Clin Med. 2026 Feb 6;15(3):1291. doi: 10.3390/jcm15031291 (PMC12898395; doi:10.3390/jcm15031291)
Supplement: Supplementary file 1 [file jcm-15-01291-s001.zip › Supplementary Table 4.pdf]

Supplementary Table 4: Cronbach's Alpha for TAPES-R Subscales

**TAPES-R Subscale**		**Cronbach's α**
**Psychosocial adjustment**	**General Adjustment**	0.855
	**Social Adjustment**	0.934
	**Adjustment to Limitation**	0.816
	**Total score**	0.848
**Activity Restriction (out of 20)**		0.912
**Satisfaction with prosthesis**	**Aesthetic Satisfaction**	0.955
	**Functional Satisfaction**	0.871
	**Total score**	0.880
